# Crystal structure of 2-(3,4-di­meth­oxy­phen­yl)-3-hy­droxy-4*H*-chromen-4-one

**DOI:** 10.1107/S1600536814018212

**Published:** 2014-08-13

**Authors:** Jin Sil Yoo, Yoongho Lim, Dongsoo Koh

**Affiliations:** a Department of Applied Chemistry, Dongduk Women’s University, Seoul 136-714, Republic of Korea; b Division of Bioscience and Biotechnology, BMIC, Konkuk University, Seoul 143-701, Republic of Korea

**Keywords:** crystal structure, 4*H*-chromen-4-one, biological properties, flavonols, natural products

## Abstract

In the title compound, C_17_H_14_O_5_, the dimeth­oxy-substituted benzene ring is twisted relative to the 4*H*-chromenon skeleton (r.m.s. deviation = 0.015 Å) by 5.2 (4)°. The C atoms of the meth­oxy groups lie close to the plane of their attached benzene ring [deviations = 0.036 (3) and 0.290 (3)Å for the *meta* and *para* substituents, respectively]. An intra­molecular O—H⋯O hydrogen bond closes an *S*(5) ring. In the cystal, inversion dimers linked by pairs of O—H⋯O hydrogen bonds generate *R*_2_^2^(10) loops and C—H⋯O inter­actions connect the dimers into [010] chains.

## Related literature   

For the syntheses and biological properties of flavonols, see: Lee *et al.* (2014 ▶); Singh *et al.* (2014 ▶); Dias *et al.* (2013 ▶); Yong *et al.* (2013 ▶). For flavonols in natural products, see: Bendaikha *et al.* (2014 ▶); Prescott *et al.* (2013 ▶). For related structures, see: Marciniec *et al.* (2013 ▶); Serdiuk *et al.* (2013 ▶); Yu *et al.* (2006 ▶).
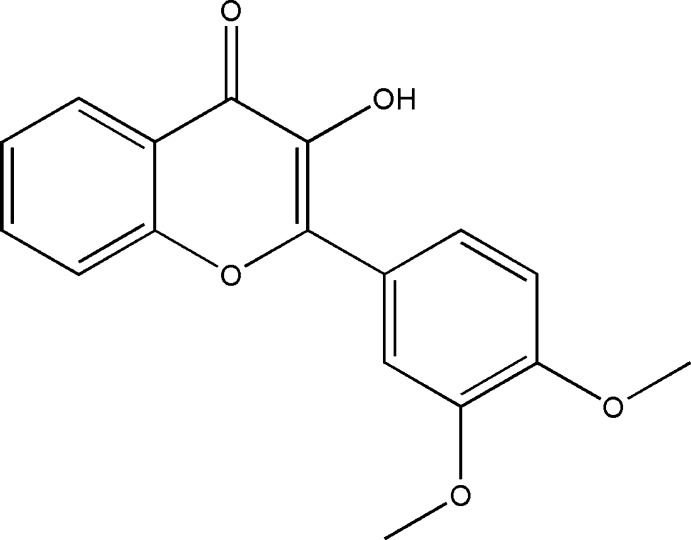


## Experimental   

### Crystal data  

C_17_H_14_O_5_
*M* *_r_* = 298.28
Monoclinic, 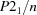

*a* = 8.2009 (7) Å
*b* = 9.2917 (8) Å
*c* = 18.2684 (15) Å
β = 96.322 (2)°
*V* = 1383.6 (2) Å^3^
*Z* = 4
Mo *K*α radiation
μ = 0.11 mm^−1^
*T* = 200 K
0.31 × 0.18 × 0.09 mm

### Data collection  

Bruker SMART CCD area-detector diffractometer
9945 measured reflections
3442 independent reflections
2438 reflections with *I* > 2σ(*I*)
*R*_int_ = 0.033

### Refinement  

*R*[*F* ^2^ > 2σ(*F* ^2^)] = 0.053
*wR*(*F* ^2^) = 0.204
*S* = 1.20
3442 reflections
202 parameters
H-atom parameters constrained
Δρ_max_ = 0.41 e Å^−3^
Δρ_min_ = −0.56 e Å^−3^

### 

Data collection: *SMART* (Bruker, 2000 ▶); cell refinement: *SAINT* (Bruker, 2000 ▶); data reduction: *SAINT*; program(s) used to solve structure: *SHELXS97* (Sheldrick, 2008 ▶); program(s) used to refine structure: *SHELXL97* (Sheldrick, 2008 ▶); molecular graphics: *SHELXTL* (Sheldrick, 2008 ▶); software used to prepare material for publication: *SHELXTL*.

## Supplementary Material

Crystal structure: contains datablock(s) I, New_Global_Publ_Block. DOI: 10.1107/S1600536814018212/hb7265sup1.cif

Structure factors: contains datablock(s) I. DOI: 10.1107/S1600536814018212/hb7265Isup2.hkl

**Fig. 1.**
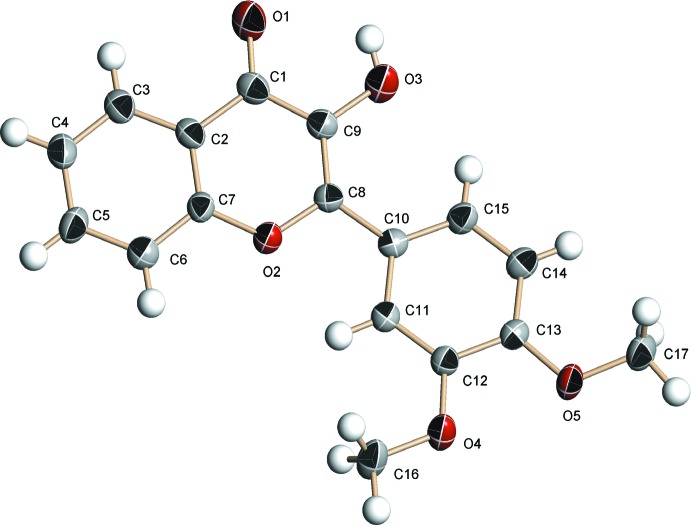
Molecular structure of the title compound, showing displacement ellipsoids drawn at the 50% probability level.

. DOI: 10.1107/S1600536814018212/hb7265fig1.tif
Mol­ecular structure of the title compound, showing displacement ellipsoids drawn at the 50% probability level.

**Fig. 2.**
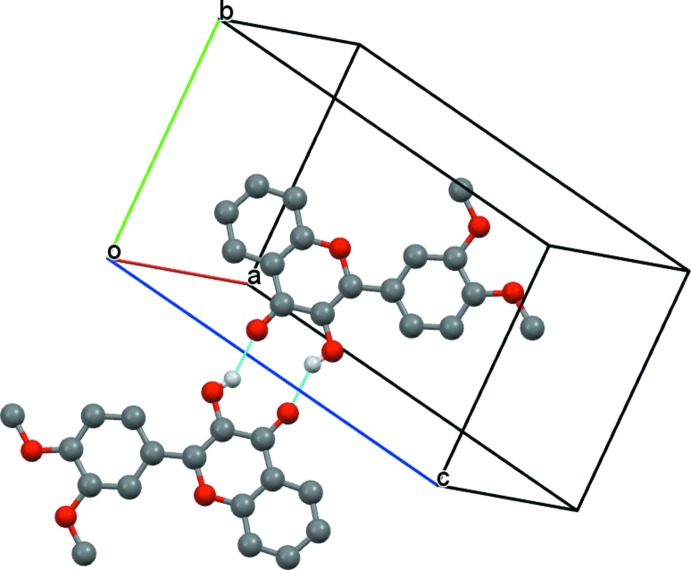
Part of the crystal structure with intermolecular O—H···O hydrogen bonds shown as dashed lines

. DOI: 10.1107/S1600536814018212/hb7265fig2.tif
Part of the crystal structure with inter­molecular O—H⋯O hydrogen bonds shown as dashed lines

CCDC reference: 1018484

Additional supporting information:  crystallographic information; 3D view; checkCIF report

## Figures and Tables

**Table 1:** Hydrogen-bond geometry (Å, °)

*D*—H⋯*A*	*D*—H	H⋯*A*	*D*⋯*A*	*D*—H⋯*A*
O3—H3*A*⋯O1	0.84	2.26	2.710 (3)	113
O3—H3*A*⋯O1^i^	0.84	1.96	2.719 (3)	150
C17—H17*A*⋯O4^ii^	0.98	2.56	3.283 (3)	130

Symmetry codes: (i) 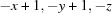; (ii) 
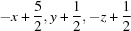
.

## supplementary crystallographic information

### S2.1. Synthesis and crystallization

Chalcone (1 mmol, 284 mg) was suspended in 15 ml of MeOH / THF (2:1), and 0.5 mL NaOH (30% aq.) was added to produce a red solution, which was cooled to 0°C. To this reaction mixture, was added 1 ml H_2_O_2_ (32% aq.) and the solution was stirred for 2h at room temperature. The resulting solution was poured into water (100 ml) and was acidified with 3M HCl. The pale yellow precipitate obtained was filtered and washed with ethanol give the titled compound (57%). Recrystallization in the ethanol solvent gave orange blocks of the title compound (mp: 475-476K)

### S2.2. Refinement

The H atoms were placed at calculated positions and refined as riding with C–H = 0.95 A [*U*iso(H) = 1.2 *U*eq(C)].

### S3. Results and discussion

Flavonoids are one of secondary metabolites in plants with C6—C3—C6 skeleton, which include flavones, flavonols, chalcones and isoflavones. Variety of flanonols have been isolated from natural sources and syntheized (Bendaikha *et al.* 2014; Prescott *et al.* 2013), because they have shown wide spectrum of biological activities (Lee *et al.* 2014; Dias *et al.* 2013). Inspired by the important biological activities of flavonols, our research project has been focused on development of novel flavonols which show broad range of biological activities. Because it has been well established that the presence and position of hy­droxy and meth­oxy substituents plays an important role in determining the biological activity of flavonoids (Singh *et al.* 2014), the title compound was synthesized and its crystal structure was determined. A starting material, chalcone, (E)-3-(3,4-di­meth­oxy­phenyl)-1-(2-hy­droxy­phenyl)­prop-2-en-1-one, was prepared by the previously reported methods (Yong *et al.* 2013). Flavonol was obtained by oxidative cyclization of the chalcone with H_2_O_2_ in alkaline methanol medium (Lee *et al.* 2014).

In the title compound, C_17_H_14_O_5_, di­meth­oxy substituted benzene ring is twisted relative to 4*H*-chromenon skeleton by 5.2 (4)^o^. The meth­oxy groups at C12 and C13 are tilted from benzene ring by 2.7 (3)^o^ and 8.9 (4)^o^, respectively. In the crystal, pairs of O—H—O hydrogen bonds form inversion dimer with graph-set notation *R*_2_^2^(10) (Marciniec *et al.* 2013). In addition, each molecule contains intra­molecular O—H—O hydrogen bond with a *S*(5) motif. Examples of structures of flavonols have been published (Serdiuk *et al.*, 2013; Yu *et al.*, 2006).

### Crystal data

**Table:**

C_17_H_14_O_5_	*F*(000) = 624
*M**_r_* = 298.28	*D*_x_ = 1.432 Mg m^−^^3^
Monoclinic, *P*2_1_/*n*	Mo *K*α radiation, λ = 0.71073 Å
Hall symbol: -P 2yn	Cell parameters from 5723 reflections
*a* = 8.2009 (7) Å	θ = 2.2–28.3°
*b* = 9.2917 (8) Å	µ = 0.11 mm^−^^1^
*c* = 18.2684 (15) Å	*T* = 200 K
β = 96.322 (2)°	Block, orange
*V* = 1383.6 (2) Å^3^	0.31 × 0.18 × 0.09 mm
*Z* = 4	

### Data collection

**Table:**

Bruker SMART CCD area-detector diffractometer	2438 reflections with *I* > 2σ(*I*)
Radiation source: fine-focus sealed tube	*R*_int_ = 0.033
Graphite monochromator	θ_max_ = 28.3°, θ_min_ = 2.2°
phi and ω scans	*h* = −10→8
9945 measured reflections	*k* = −12→10
3442 independent reflections	*l* = −24→23

### Refinement

**Table:**

Refinement on *F*^2^	Primary atom site location: structure-invariant direct methods
Least-squares matrix: full	Secondary atom site location: difference Fourier map
*R*[*F*^2^ > 2σ(*F*^2^)] = 0.053	Hydrogen site location: inferred from neighbouring sites
*wR*(*F*^2^) = 0.204	H-atom parameters constrained
*S* = 1.20	*w* = 1/[σ^2^(*F*_o_^2^) + (0.0768*P*)^2^ + 1.2451*P*] where *P* = (*F*_o_^2^ + 2*F*_c_^2^)/3
3442 reflections	(Δ/σ)_max_ < 0.001
202 parameters	Δρ_max_ = 0.41 e Å^−^^3^
0 restraints	Δρ_min_ = −0.56 e Å^−^^3^

### Special details

**Table:**

Geometry. All e.s.d.'s (except the e.s.d. in the dihedral angle between two l.s. planes) are estimated using the full covariance matrix. The cell e.s.d.'s are taken into account individually in the estimation of e.s.d.'s in distances, angles and torsion angles; correlations between e.s.d.'s in cell parameters are only used when they are defined by crystal symmetry. An approximate (isotropic) treatment of cell e.s.d.'s is used for estimating e.s.d.'s involving l.s. planes.
Refinement. Refinement of *F*^2^ against ALL reflections. The weighted *R*-factor *wR* and goodness of fit *S* are based on *F*^2^, conventional *R*-factors *R* are based on *F*, with *F* set to zero for negative *F*^2^. The threshold expression of *F*^2^ > σ(*F*^2^) is used only for calculating *R*-factors(gt) *etc*. and is not relevant to the choice of reflections for refinement. *R*-factors based on *F*^2^ are statistically about twice as large as those based on *F*, and *R*- factors based on ALL data will be even larger.

### Fractional atomic coordinates and isotropic or equivalent isotropic displacement parameters (Å^2^)

**Table:**

	*x*	*y*	*z*	*U*_iso_*/*U*_eq_	
O1	0.4617 (3)	0.3933 (2)	−0.06661 (11)	0.0482 (5)	
C1	0.5311 (3)	0.2734 (3)	−0.06091 (14)	0.0359 (5)	
C2	0.4995 (3)	0.1608 (3)	−0.11530 (13)	0.0331 (5)	
C3	0.3917 (3)	0.1776 (3)	−0.17952 (14)	0.0376 (6)	
H3	0.3373	0.2669	−0.1893	0.045*	
C4	0.3639 (3)	0.0662 (3)	−0.22855 (14)	0.0418 (6)	
H4	0.2905	0.0787	−0.2721	0.050*	
C5	0.4433 (3)	−0.0657 (3)	−0.21466 (14)	0.0415 (6)	
H5	0.4236	−0.1426	−0.2488	0.050*	
C6	0.5503 (3)	−0.0846 (3)	−0.15152 (14)	0.0366 (5)	
H6	0.6038	−0.1743	−0.1417	0.044*	
C7	0.5784 (3)	0.0295 (3)	−0.10267 (12)	0.0314 (5)	
O2	0.6861 (2)	0.00521 (17)	−0.04197 (9)	0.0324 (4)	
C8	0.7237 (3)	0.1100 (2)	0.00988 (12)	0.0294 (5)	
C9	0.6471 (3)	0.2409 (3)	0.00173 (13)	0.0328 (5)	
O3	0.6804 (2)	0.34504 (19)	0.05317 (11)	0.0446 (5)	
H3A	0.6195	0.4164	0.0425	0.067*	
C10	0.8434 (3)	0.0586 (2)	0.06893 (12)	0.0299 (5)	
C11	0.8933 (3)	−0.0875 (2)	0.06779 (12)	0.0302 (5)	
H11	0.8517	−0.1475	0.0280	0.036*	
C12	1.0008 (3)	−0.1431 (2)	0.12337 (12)	0.0302 (5)	
C13	1.0641 (3)	−0.0565 (3)	0.18262 (12)	0.0309 (5)	
C14	1.0198 (3)	0.0875 (3)	0.18304 (14)	0.0359 (5)	
H14	1.0646	0.1479	0.2221	0.043*	
C15	0.9108 (3)	0.1445 (3)	0.12696 (14)	0.0348 (5)	
H15	0.8818	0.2434	0.1282	0.042*	
O4	1.0533 (2)	−0.28329 (18)	0.12628 (9)	0.0386 (4)	
C16	0.9847 (4)	−0.3755 (3)	0.06895 (16)	0.0467 (7)	
H16A	0.8652	−0.3785	0.0688	0.070*	
H16B	1.0296	−0.4726	0.0770	0.070*	
H16C	1.0119	−0.3389	0.0215	0.070*	
O5	1.1652 (2)	−0.12376 (19)	0.23612 (9)	0.0387 (4)	
C17	1.2109 (4)	−0.0443 (3)	0.30216 (14)	0.0456 (7)	
H17A	1.2667	0.0445	0.2903	0.068*	
H17B	1.2849	−0.1026	0.3360	0.068*	
H17C	1.1124	−0.0204	0.3256	0.068*	

### Atomic displacement parameters (Å^2^)

**Table:**

	*U*^11^	*U*^22^	*U*^33^	*U*^12^	*U*^13^	*U*^23^
O1	0.0552 (12)	0.0343 (10)	0.0526 (11)	0.0138 (8)	−0.0047 (9)	0.0018 (8)
C1	0.0362 (12)	0.0309 (12)	0.0411 (13)	0.0041 (10)	0.0072 (10)	0.0040 (10)
C2	0.0320 (12)	0.0341 (12)	0.0337 (11)	0.0015 (9)	0.0064 (9)	0.0060 (9)
C3	0.0325 (12)	0.0419 (13)	0.0386 (13)	0.0033 (10)	0.0049 (10)	0.0057 (10)
C4	0.0348 (13)	0.0535 (16)	0.0364 (12)	−0.0016 (11)	0.0010 (10)	0.0084 (11)
C5	0.0385 (13)	0.0535 (16)	0.0310 (12)	−0.0045 (12)	−0.0021 (10)	−0.0052 (11)
C6	0.0367 (13)	0.0341 (12)	0.0387 (12)	0.0027 (10)	0.0032 (10)	−0.0021 (10)
C7	0.0275 (11)	0.0383 (12)	0.0284 (10)	−0.0015 (9)	0.0024 (8)	0.0025 (9)
O2	0.0334 (9)	0.0288 (8)	0.0338 (8)	0.0034 (6)	−0.0010 (7)	−0.0029 (6)
C8	0.0288 (11)	0.0274 (11)	0.0323 (11)	0.0008 (8)	0.0043 (9)	−0.0005 (8)
C9	0.0352 (12)	0.0279 (11)	0.0355 (12)	0.0019 (9)	0.0047 (9)	−0.0007 (9)
O3	0.0531 (12)	0.0278 (9)	0.0499 (11)	0.0098 (8)	−0.0076 (9)	−0.0076 (8)
C10	0.0287 (11)	0.0295 (11)	0.0319 (11)	0.0007 (9)	0.0056 (9)	−0.0028 (9)
C11	0.0295 (11)	0.0306 (11)	0.0305 (11)	0.0011 (9)	0.0033 (9)	−0.0020 (9)
C12	0.0307 (11)	0.0290 (11)	0.0304 (11)	0.0017 (9)	0.0019 (9)	−0.0008 (9)
C13	0.0289 (11)	0.0325 (12)	0.0312 (11)	−0.0004 (9)	0.0025 (9)	−0.0036 (9)
C14	0.0362 (12)	0.0346 (12)	0.0366 (12)	−0.0012 (10)	0.0020 (10)	−0.0068 (10)
C15	0.0348 (12)	0.0293 (11)	0.0392 (12)	0.0016 (9)	−0.0010 (10)	−0.0033 (9)
O4	0.0478 (10)	0.0286 (9)	0.0364 (9)	0.0067 (7)	−0.0086 (7)	−0.0043 (7)
C16	0.0587 (17)	0.0295 (12)	0.0469 (15)	0.0069 (12)	−0.0167 (13)	−0.0093 (11)
O5	0.0425 (10)	0.0384 (9)	0.0321 (8)	0.0018 (8)	−0.0089 (7)	−0.0028 (7)
C17	0.0534 (16)	0.0455 (15)	0.0351 (13)	−0.0058 (12)	−0.0086 (11)	−0.0064 (11)

### Geometric parameters (Å, º)

**Table:**

O1—C1	1.250 (3)	C10—C15	1.392 (3)
C1—C9	1.437 (3)	C10—C11	1.418 (3)
C1—C2	1.447 (4)	C11—C12	1.370 (3)
C2—C7	1.388 (3)	C11—H11	0.9500
C2—C3	1.398 (3)	C12—O4	1.371 (3)
C3—C4	1.371 (4)	C12—C13	1.401 (3)
C3—H3	0.9500	C13—O5	1.362 (3)
C4—C5	1.397 (4)	C13—C14	1.387 (3)
C4—H4	0.9500	C14—C15	1.388 (3)
C5—C6	1.381 (3)	C14—H14	0.9500
C5—H5	0.9500	C15—H15	0.9500
C6—C7	1.388 (3)	O4—C16	1.420 (3)
C6—H6	0.9500	C16—H16A	0.9800
C7—O2	1.359 (3)	C16—H16B	0.9800
O2—C8	1.370 (3)	C16—H16C	0.9800
C8—C9	1.369 (3)	O5—C17	1.429 (3)
C8—C10	1.457 (3)	C17—H17A	0.9800
C9—O3	1.356 (3)	C17—H17B	0.9800
O3—H3A	0.8400	C17—H17C	0.9800
			
O1—C1—C9	120.6 (2)	C11—C10—C8	118.4 (2)
O1—C1—C2	122.8 (2)	C12—C11—C10	120.9 (2)
C9—C1—C2	116.6 (2)	C12—C11—H11	119.6
C7—C2—C3	118.5 (2)	C10—C11—H11	119.6
C7—C2—C1	118.5 (2)	C11—C12—O4	124.1 (2)
C3—C2—C1	123.0 (2)	C11—C12—C13	120.6 (2)
C4—C3—C2	120.5 (2)	O4—C12—C13	115.3 (2)
C4—C3—H3	119.7	O5—C13—C14	125.3 (2)
C2—C3—H3	119.7	O5—C13—C12	115.8 (2)
C3—C4—C5	120.2 (2)	C14—C13—C12	118.9 (2)
C3—C4—H4	119.9	C13—C14—C15	120.8 (2)
C5—C4—H4	119.9	C13—C14—H14	119.6
C6—C5—C4	120.2 (3)	C15—C14—H14	119.6
C6—C5—H5	119.9	C14—C15—C10	120.9 (2)
C4—C5—H5	119.9	C14—C15—H15	119.6
C5—C6—C7	119.0 (2)	C10—C15—H15	119.6
C5—C6—H6	120.5	C12—O4—C16	116.58 (18)
C7—C6—H6	120.5	O4—C16—H16A	109.5
O2—C7—C6	116.4 (2)	O4—C16—H16B	109.5
O2—C7—C2	122.0 (2)	H16A—C16—H16B	109.5
C6—C7—C2	121.5 (2)	O4—C16—H16C	109.5
C7—O2—C8	121.53 (18)	H16A—C16—H16C	109.5
C9—C8—O2	119.4 (2)	H16B—C16—H16C	109.5
C9—C8—C10	129.5 (2)	C13—O5—C17	116.8 (2)
O2—C8—C10	111.13 (19)	O5—C17—H17A	109.5
O3—C9—C8	120.2 (2)	O5—C17—H17B	109.5
O3—C9—C1	117.8 (2)	H17A—C17—H17B	109.5
C8—C9—C1	121.9 (2)	O5—C17—H17C	109.5
C9—O3—H3A	109.5	H17A—C17—H17C	109.5
C15—C10—C11	117.9 (2)	H17B—C17—H17C	109.5
C15—C10—C8	123.7 (2)		
			
O1—C1—C2—C7	−177.7 (2)	C2—C1—C9—O3	179.3 (2)
C9—C1—C2—C7	1.8 (3)	O1—C1—C9—C8	179.3 (2)
O1—C1—C2—C3	1.5 (4)	C2—C1—C9—C8	−0.2 (4)
C9—C1—C2—C3	−179.1 (2)	C9—C8—C10—C15	−5.2 (4)
C7—C2—C3—C4	0.5 (4)	O2—C8—C10—C15	176.3 (2)
C1—C2—C3—C4	−178.7 (2)	C9—C8—C10—C11	174.2 (2)
C2—C3—C4—C5	0.0 (4)	O2—C8—C10—C11	−4.2 (3)
C3—C4—C5—C6	0.0 (4)	C15—C10—C11—C12	2.0 (3)
C4—C5—C6—C7	−0.5 (4)	C8—C10—C11—C12	−177.5 (2)
C5—C6—C7—O2	−179.3 (2)	C10—C11—C12—O4	179.4 (2)
C5—C6—C7—C2	1.0 (4)	C10—C11—C12—C13	−0.1 (3)
C3—C2—C7—O2	179.3 (2)	C11—C12—C13—O5	177.7 (2)
C1—C2—C7—O2	−1.5 (3)	O4—C12—C13—O5	−1.8 (3)
C3—C2—C7—C6	−1.0 (4)	C11—C12—C13—C14	−1.8 (3)
C1—C2—C7—C6	178.2 (2)	O4—C12—C13—C14	178.6 (2)
C6—C7—O2—C8	179.8 (2)	O5—C13—C14—C15	−177.6 (2)
C2—C7—O2—C8	−0.5 (3)	C12—C13—C14—C15	1.9 (4)
C7—O2—C8—C9	2.1 (3)	C13—C14—C15—C10	−0.1 (4)
C7—O2—C8—C10	−179.22 (19)	C11—C10—C15—C14	−1.9 (4)
O2—C8—C9—O3	178.8 (2)	C8—C10—C15—C14	177.6 (2)
C10—C8—C9—O3	0.5 (4)	C11—C12—O4—C16	−2.7 (4)
O2—C8—C9—C1	−1.8 (4)	C13—C12—O4—C16	176.9 (2)
C10—C8—C9—C1	179.9 (2)	C14—C13—O5—C17	8.9 (3)
O1—C1—C9—O3	−1.3 (4)	C12—C13—O5—C17	−170.6 (2)

### Hydrogen-bond geometry (Å, º)

**Table:**

*D*—H···*A*	*D*—H	H···*A*	*D*···*A*	*D*—H···*A*
O3—H3*A*···O1	0.84	2.26	2.710 (3)	113
O3—H3*A*···O1^i^	0.84	1.96	2.719 (3)	150
C17—H17*A*···O4^ii^	0.98	2.56	3.283 (3)	130

Symmetry codes: (i) −*x*+1, −*y*+1, −*z*; (ii) −*x*+5/2, *y*+1/2, −*z*+1/2.
