# Supplementary figures and images for: Crystal structure of 2-(3,4-di­meth­oxy­phen­yl)-3-hy­droxy-4H-chromen-4-one
Source: Acta Crystallogr Sect E Struct Rep Online. 2014 Aug 13;70(Pt 9):o999–o1000. doi: 10.1107/S1600536814018212 (PMC4186158; doi:10.1107/S1600536814018212)

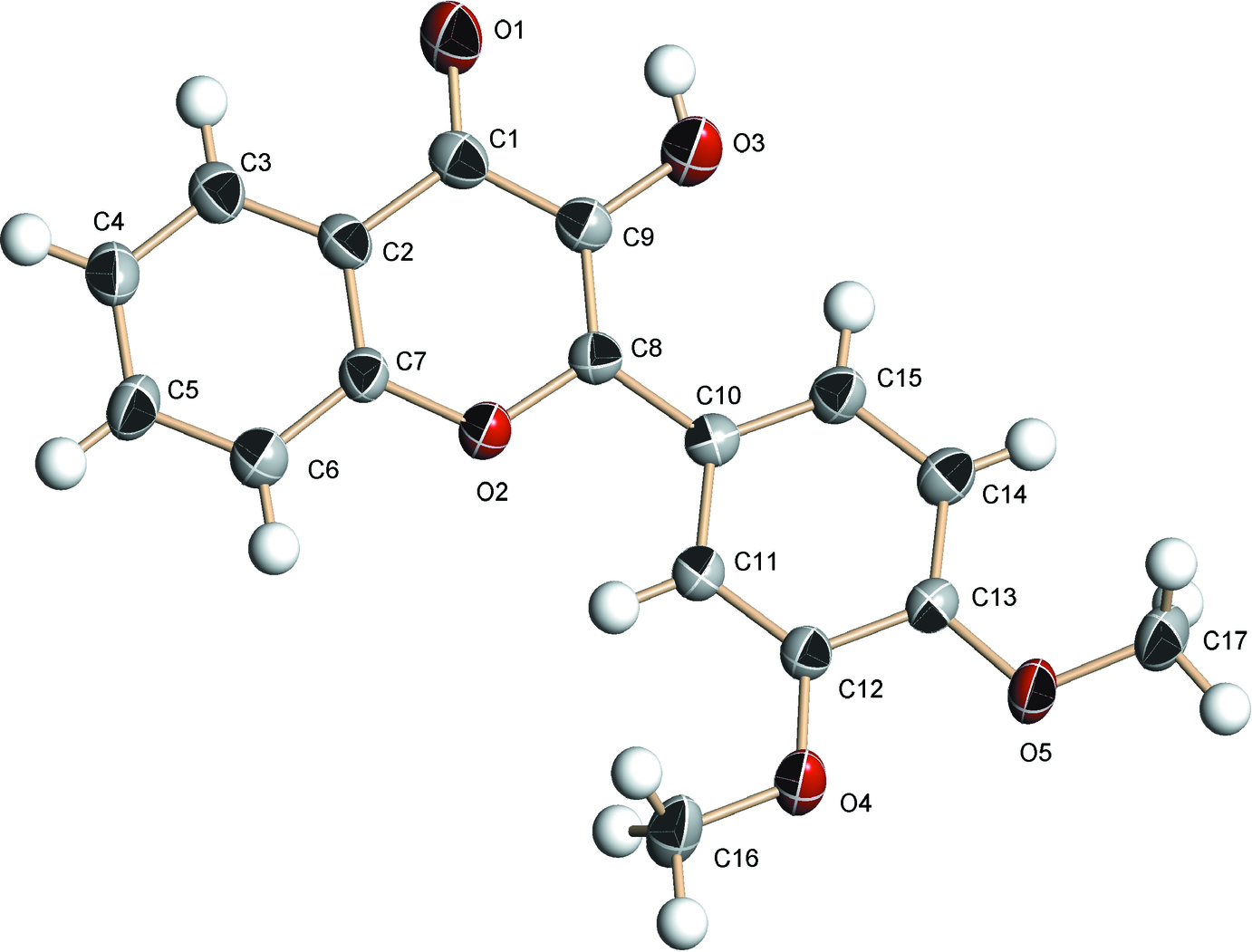

Supplement: Supplementary file 3 [file e-70-0o999-fig1.tif]

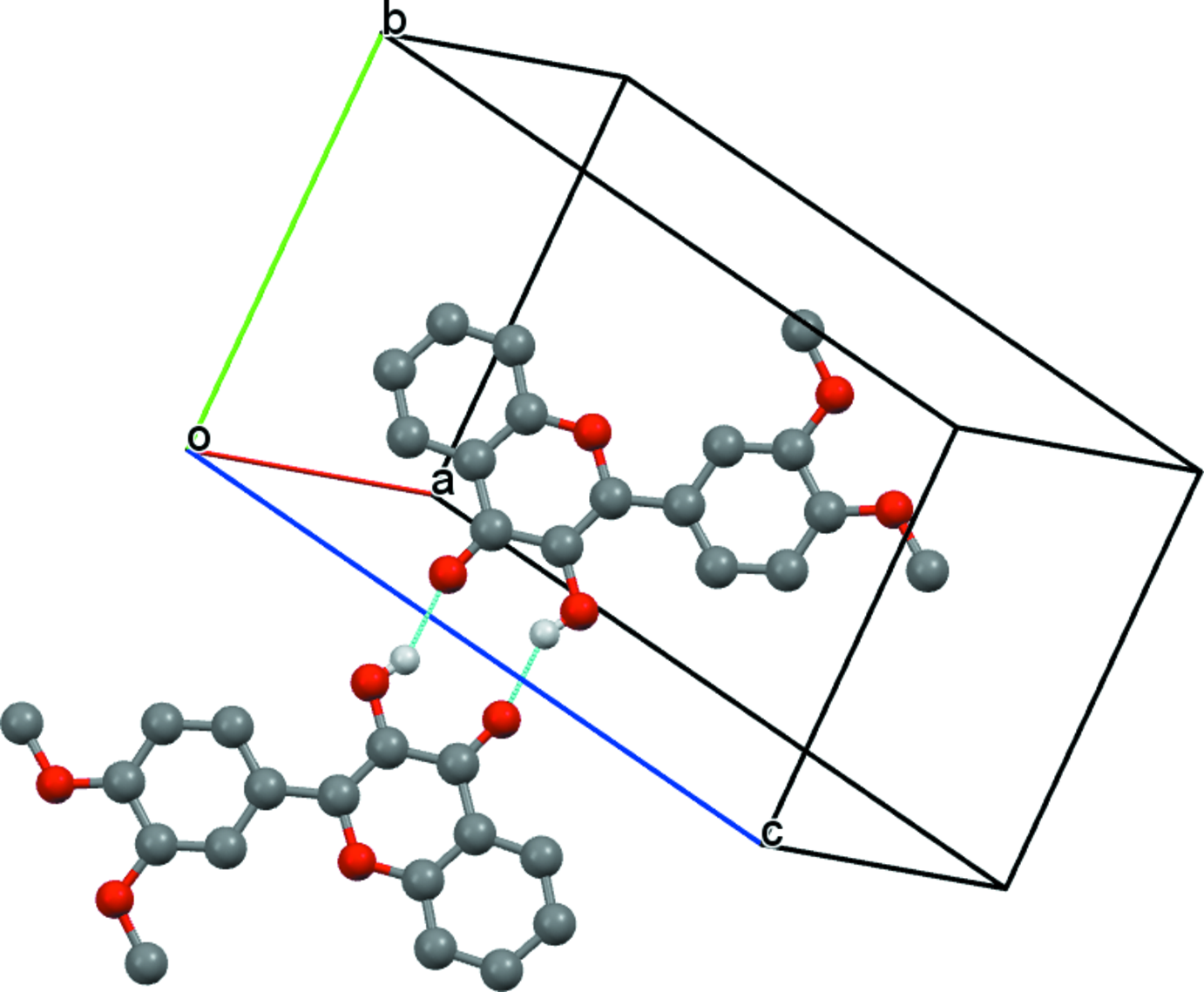

Supplement: Supplementary file 4 [file e-70-0o999-fig2.tif]
